# EIF4A3-induced circ_0084615 contributes to the progression of colorectal cancer via miR-599/ONECUT2 pathway

**DOI:** 10.1186/s13046-021-02029-y

**Published:** 2021-07-12

**Authors:** Zhipeng Jiang, Qinwen Tai, Xiaojun Xie, Zehui Hou, Wei Liu, Zhuomin Yu, Zhiqiang Liang, Shuang Chen

**Affiliations:** 1 Department of Gastrointestinal Surgery, Guangdong Institute of Gastroenterology, Guangdong Provincial Key Laboratory of Colorectal and Pelvic Floor Diseases, The Sixth Affiliated Hospital of Sun Yat-sen University, Supported by National Key Clinical Discipline, No.26 YuanCun 2nd Heng Road, Guangdong Province 510655 Guangzhou City, China; 2 Department of General Surgery, Shenzhen Hospital of Southern Medical University, Shenzhen City, Guangdong Province China; 3 Department of General Surgery, The First Affiliated Hospital of Shantou University Medical College, Shantou City, Guangdong Province China

**Keywords:** Colorectal cancer, circ_0084615, miR-599, ONECUT2, EIF4A3

## Abstract

**Background:**

Colorectal cancer (CRC) is a common malignant tumor. Circular RNAs (circRNAs) have been reported to take part in the progression of CRC. However, the functions of circ_0084615 in CRC development are still undefined. In this study, we aimed to explore the functions and underlying mechanisms of circ_0084615 in CRC.

**Methods:**

qRT-PCR, western blot assay and IHC assay were utilized for the levels of circ_0084615, miR-599, ONECUT2 or EIF4A3. 5-ethynyl-2’-deoxyuridine (EdU) assay and colony formation assay were conducted for cell proliferation ability. Wound-healing assay and transwell assay were applied to evaluate cell migration and invasion. Tube formation assay was used to analyze angiogenesis ability. RNA immunoprecipitation (RIP) assay, RNA pull down assay and dual-luciferase reporter assay were used to analyze the relationships of circ_0084615, miR-599, ONECUT2 and EIF4A3. Murine xenograft model assay was employed for the role of circ_0084615 *in vivo*.

**Results:**

Circ_0084615 was elevated in CRC tissues and was linked to TNM stages, lymph node metastasis, differentiation and overall survival rate. Circ_0084615 knockdown inhibited CRC cell proliferation, migration, invasion and angiogenesis *in vitro* and hampered tumorigenesis *in vivo*. Circ_0084615 sponged miR-599 and miR-599 inhibition reversed circ_0084615 knockdown-mediated effects on CRC cell growth, motility and angiogenesis. ONECUT2 was identified as the target gene of miR-599. ONECUT2 overexpression recovered the effects of miR-599 on CRC malignant behaviors. Additionally, EIF4A3 induced circ_0084615 expression.

**Conclusions:**

EIF4A3-induced circ_0084615 played an oncogenic role in CRC development via miR-599/ONECUT2 axis.

**Supplementary Information:**

The online version contains supplementary material available at 10.1186/s13046-021-02029-y.

## Background

Colorectal cancer (CRC) is a commen cancer that threatens people’s health and is highly linked to cancer-associated mortality [1, 2]. The treatment options for CRC include surgery, chemotherapy, radiotherapy and adjuvant therapy [3]. However, tumor metastasis is the main reason for the death in CRC patients and the prognosis of patients with metastasis CRC is dismal [4]. Molecular markers can be used as therapeutic targets for CRC [5]. Consequently, it is imperative to explore the mechanism of CRC progress and identify novel targets.

The non-coding circular RNAs (circRNAs) are commonly expressed in various tumor cell types and modulate tumor progression [6, 7]. More importantly, circRNAs have been recognized as microRNA (miRNA) sponges to alter gene expression to modulate the carcinogenesis of human cancers [8]. For example, circRAE1 exacerbated the motility of CRC via elevating TYRO3 through sponging miR-338-3p [9]. Circ_0038646 facilitated the growth and migration of CRC cells through modulating miR-331-3p/GRIK3 axis [10]. These reports indicated the oncogenic role of circRNAs in CRC. Circ_0084615 derived from aspartate beta-hydroxylase (ASPH) and was found to be upregulated in CRC via GEO databases. However, the precise functions of circ_0084615 in CRC are barely known.

MiRNAs are a flock of non-coding RNAs harboring 18–22 nucleotides and are implicated in tumor progression [11]. Among these, miR-599 was demonstrated to serve as a suppressor in diverse human cancers, such as bladder urothelial carcinoma [12], anaplastic thyroid carcinoma [13], gastric cancer [14] and glioma [15]. Furthermore, miR-599 participated in CRC cell progression via lncRNA MCM3AP-AS1/miR-599/ARPP19 axis [16]. However, the mechanisms mediated by miR-599 in PC remain largely unknown.

One cut homeobox 2 (ONECUT2, also named OC2) is related to the proliferation, motility and differentiation of tumor cells [17]. In CRC, ONECUT2 influenced the growth, invasion and epithelial-mesenchymal transition (EMT) via acting as the target of miR-429 [18]. Based on bioinformatics analysis, miR-599 was found to contain the binding sites of circ_0084615 and ONECUT2. However, the relationships among circ_0084615, miR-599 and ONECUT2 are not clear.

In the present research, the expression profiles of circ_0084615, miR-599 and ONECUT2 in CRC were determined and their relationships in CRC development were revealed.

## Materials and methods

### Tissue specimens acquisition

 CRC and nearby non-tumor tissues were acquired from CRC patients (*n* = 54) at The Sixth Affiliated Hospital of Sun Yat-sen University after the work was authorized by the Ethics Committee of The Sixth Affiliated Hospital of Sun Yat-sen University and written informed consents were provided by the patients. The tissues were preserved at -80 °C until use. The correlation of circ_0083615 expression with the clinicopathologic features in CRC patients were exhibited in Table 1.

**Table 1:** Correlation of the expression of circ_0083615 with clinicopathologic features in CRC patients

Parameters		circ_0083615 expression		*p*-value
	N=54	High N=27	Low N=27	
Age,years				
<60	25	11	14	0.413
≥60	29	16	13	
Gender				
Male	32	18	14	0.268
Female	22	9	13	
Tumor size				
<3	35	16	19	0.393
≥3	19	11	8	
Lymph node metastasis				
No	33	11	22	0.002^***^
Yes	21	16	5	
Differentiation				
Well/Moderate	34	11	23	<0.001^***^
Poor	22	18	4	
TNM stage				
I + II	29	10	19	0.014^*^
III	25	17	8	
Chemotherapy				
sensitive	25	11	14	0.413
resistant	29	16	13	

### Cell culture

The American Type Culture Collection (ATCC, Manassas, VA, USA) offered FHC, SW620, DLD1, SW480 and HT-29 cells. RPMI1640 (Invitrogen, Carlsbad, CA, USA) mixed with 10 % fetal bovine serum (FBS; Invitrogen) and 1 % penicillin-streptomycin (Invitrogen) was utilized to culture the cells at a cultivation environment of 5 % CO_2_ and 37 °C.

### Bioinformatic analysis

The differentially expressed circRNAs were analyzed by GSE126094, GSE138589 and GSE142837. The binding sites between circ_0084615 and miR-599 were analyzed by starbase (http://starbase.sysu.edu.cn/starbase2/) and circinteractome (https://circinteractome.irp.nia.nih.gov/). Then binding sites between ONECUT2 and miR-599 were analyzed by starbase and miRDB (http://mirdb.org/). The binding between EIF4A3 and ASPH was analyzed via circinteractome (https://circinteractome.irp.nia.nih.gov/).

### Quantitative real-time polymerase chain reaction (qRT-PCR)

The RNA was extracted utilizing TRIzol (Invitrogen) and then reversely transcribed into cDNA via the usage of High Capacity cDNA Reverse Transcription Kit (Applied Biosystems, Carlsbad, CA, USA) or TaqMan miRNA assays (Applied Biosystems) and either random hexamer primers or oligo (dT)18 primers. qRT-PCR was then conducted using SYBR Green qPCR Mix (Invitrogen) and related primers (GenePharma, Shanghai, China). The primer sequences were exhibited in Table 2. The relative expression was normalized to GAPDH or U6 and computed via 2^−ΔΔCt^ method.

**Table 2:** Primers sequences used for qRT-PCR

Name		Primers for PCR (5’-3’)
hsa_circ_0084615	Forward	ACTTATCAGAGGTGCTTCAAGG
	Reverse	TGTGTCCTCCATGCTTTGTCT
hsa_circ_0040809	Forward	TGCAACAAAGTGCGATGGTG
	Reverse	CAGGTCGTGTTCCGACATCA
hsa_circ_0000467	Forward	GAGGAATAATAAAAGTACACGAGCA
	Reverse	GCAACAGGAGGATCAGACAGA
hsa_circ_0000512	Forward	AGCTTGGAACAGACTCACGG
	Reverse	ATCTCCTGCCCAGTCTGACC
ASPH	Forward	GGAACAAGCAGTATATGAACCTCT
	Reverse	ATGGTTAGGCTGGTCCTCCT
miR-599	Forward	GCCGAGGTTGTGTCAGTTT
	Reverse	CTCAACTGGTGTCGTGGAGT
ONECUT2	Forward	CCGAACACTCTTCGCCATCT
	Reverse	GCTCAGATCGTCTTGCCACT
GAPDH	Forward	GACAGTCAGCCGCATCTTCT
	Reverse	GCGCCCAATACGACCAAATC
U6	Forward	CTCGCTTCGGCAGCACA
	Reverse	AACGCTTCACGAATTTGCGT

### Actinomycin D (Act D), RNase R and subcellular fraction analysis

For Act D assay, SW620 and DLD-1 cells were exposed to Act D (Sigma-Aldrich, St. Louis, MO, USA) for indicated time points.

RNase R treatment was executed on total RNA using RNase R (Epicenter, Madison, WI, USA) for 20 min.

The cytoplasm and nucleus in SW620 and DLD-1 cells were separated with the PARIS Kit (Life Technologies, Austin,Texas, USA) following the manufacturers’ guidelines.

After the above treatments, the abundance of circ_0084615 or ASPH was determined via qRT-PCR.

### Cell transfection

Short hairpin RNA (shRNA) against circ_0084615 (sh-circ_0084615), shRNA against EIF4A3 (sh-EIF4A3) and their scramble control (sh-NC), miR-599 mimics (miR-599) and negative control (miR-NC), miR-599 inhibitors (anti-miR-599) and its control (anti-NC), ONECUT2 overexpression vector (ONECUT2) and control vector (vector), EIF4A3 overexpression vector (oe-EIF4A3) and empty control (pcDNA) were all bought from GenePharma and transfected into SW620 and DLD-1 cells via Lipofectamine 2000 (Invitrogen) referring to the protocols.

### 5-ethynyl-2’- deoxyuridine (EdU) experiment

The EdU kit (RIBOBIO, Guangzhou, China) was employed to test cell proliferation. In short, SW620 and DLD-1 cells were plated into 24-well plates and then mixed with EdU reagent. Next, cells were fixed with paraformaldehyde (Sigma-Aldrich) and mixed with 0.5 % Triton-X-100 (Sigma-Aldrich). Nuclei were dyed with EdU and DAPI (Sigma-Aldrich). The images were captured with a fluorescence microscope (Olympus, Tokyo, Japan). EdU-positive cells were counted.

### Colony formation analysis

The transfected SW620 and DLD-1 cells were kept in 6-well plates for approximately 14 days. Thereafter, the clones were stained with crystal violet (Sigma-Aldrich) and then imaged and counted.

### Wound healing assay

The transfected SW620 and DLD-1 cells were plated in 12-well plates and then a pipette tip was used to make a wound. The wound closure was measured at 0 and 24 h for the assessment of cell migration.

### Transwell assay

The chambers (Corning, Corning, NY, USA) covered with Matrigel (Corning) were utilized for cell invasion assay. Briefly, the transfected SW620 and DLD-1 cells suspended in non-serum medium were added into the upper chamber and the complete culture medium mixed with 10 % FBS (Invitrogen) was placed into the lower chamber. After incubation for 24 h, the invaded cells were stained with crystal violet (Sigma-Aldrich) for photographing and counting.

### Tube formation assay

To examine the angiogenesis ability, HUVECs (ATCC) were maintained in the 96-well plates which were covered with Matrigel (Corning). Then, the suspended SW620 and DLD-1 cells were supplemented into the wells and cultured for 8 h. After that, the tube numbers were counted under a fluorescence microscope (Olympus).

###  Western blot analysis

The tissues and cells were subjected to RIPA (Beyotime, Shanghai, China) for total protein extraction. The proteins were then separated using SDS-PAGE (Beyotime) and blotted onto PVDF membranes (Beyotime). The membranes were then blocked with 5 % slim milk, incubated with primary antibodies, followed by interaction with a secondary antibody (bs-0295 M-HRP; Bioss, Beijing, China). The proteins were detected with an ECL kit (Beyotime). The primary antibody included anti-ONECUT2 (bs-19643R; Bioss), anti-Zinc Finger E-Box Binding Homeobox 2 (anti-ZEB2; bs-20484R; Bioss), anti-E-cadherin (bs-1519R; Bioss), anti-Vimentin (bs-0756R; Bioss), anti-vascular endothelial growth factor A (VEGFA) (bs-20393R; Bioss) and β-actin (bs-0061R; Bioss).

### RNA immunoprecipitation (RIP) assay

RIP experiment was conducted with EZ-Magna RIP kit (Millipore, Billerica, MA, USA). To analyze the relationships of circ_0084615, miR-599 and ONECUT2, SW620 and DLD-1 cells were lysed in RIP buffer and incubated with magnetic beads conjugated with anti-Ago2 (bs-12450R; Bioss) or anti-IgG (bs-0297P; Bioss). Then, the RNAs were extracted from immunoprecipitated complexes and the enrichment of circ_0084615, miR-599 and ONECUT2 were detected.

To analyze to the interaction between EIF4A3 and ASPH, the lysed cells were kept with magnetic beads conjugated with anti-EIF4A3 (bs-14548R; Bioss) or anti-IgG (bs-0297P; Bioss). Next, the coprecipitated RNAs were extracted and subjected to qRT-PCR.

### RNA pull-down assay

The wild-type or mutant biotin-labeled probe miR-599 (bio-miR-599-wt or bio-miR-599-mut) or related control (bio-NC) was cultivated with streptavidin-coated magnetic beads (Invitrogen) for the generation of probe-coated beads. Then SW620 and DLD-1 cells were sonicated and kept overnight with the beads. After that, the beads-bound RNA complexes were eluted and RNAs were extracted. The enrichment of circ_0084615 and ONECUT2 was examined.

### Dual-luciferase reporter assay

 To construct luciferase reporter vectors circ_0084615-wt or ONECUT2 3’UTR-wt, the sequences of circ_0084615 or ONECUT2 3’UTR including miR-599 binding sites (GACACAA) were introduced into pmiR-REPORT™ vectors (Promega, Madison, WI, USA). Circ_0084615-wt or ONECUT2 3’UTR-wt was constructed by mutating the binding sites. Then the vectors were transfected into SW620 and DLD-1 cells in combination with miR-599 or miR-NC, followed by measurement of the luciferase intensity.

### Immunohistochemistry (IHC) assay

IHC assay was performed as previously reported [24]. The antibodies against ONECUT2 (bs-19643R), ZEB2 (bs-20484R), E-cadherin (bs-1519R), Vimentin (bs-0756R) and VEGFA (bs-20393R) were bought from Bioss.

### Murine xenograft model

Beijing Vital River Laboratory Animal Technology Co., Ltd. (Beijing, China) offered the male BALB/c nude mice. The mice were divided into 2 groups (n = 6/group) and then sh-NC or sh-circ_0084615 transfected SW620 cells were introduced into the mice. The tumor volume (1/2 (length × width^2^)) was monitored every 5 days. The mice were euthanized after 30 days and xenograft tumors were harvested for weight and IHC assay. The animal experiments were approved by the Ethics Committee of Animal Research of The Sixth Affiliated Hospital of Sun Yat-sen University.

### Statistical analysis

All experiments were executed 3 times and the data were analyzed through GraphPad Prism 7 (GraphPad, La Jolla, CA, USA). The results were exhibited as mean ± SD. The overall survival curve was analyzed through Kaplan-Meier plot and log-rank test. Difference analysis was done via Student’s *t*-test or one-way analysis of variance. *P* value less than 0.05 indicated significant.

## Results

### Circ_0084615 was upregulated in CRC tissues

**Fig. 1:**
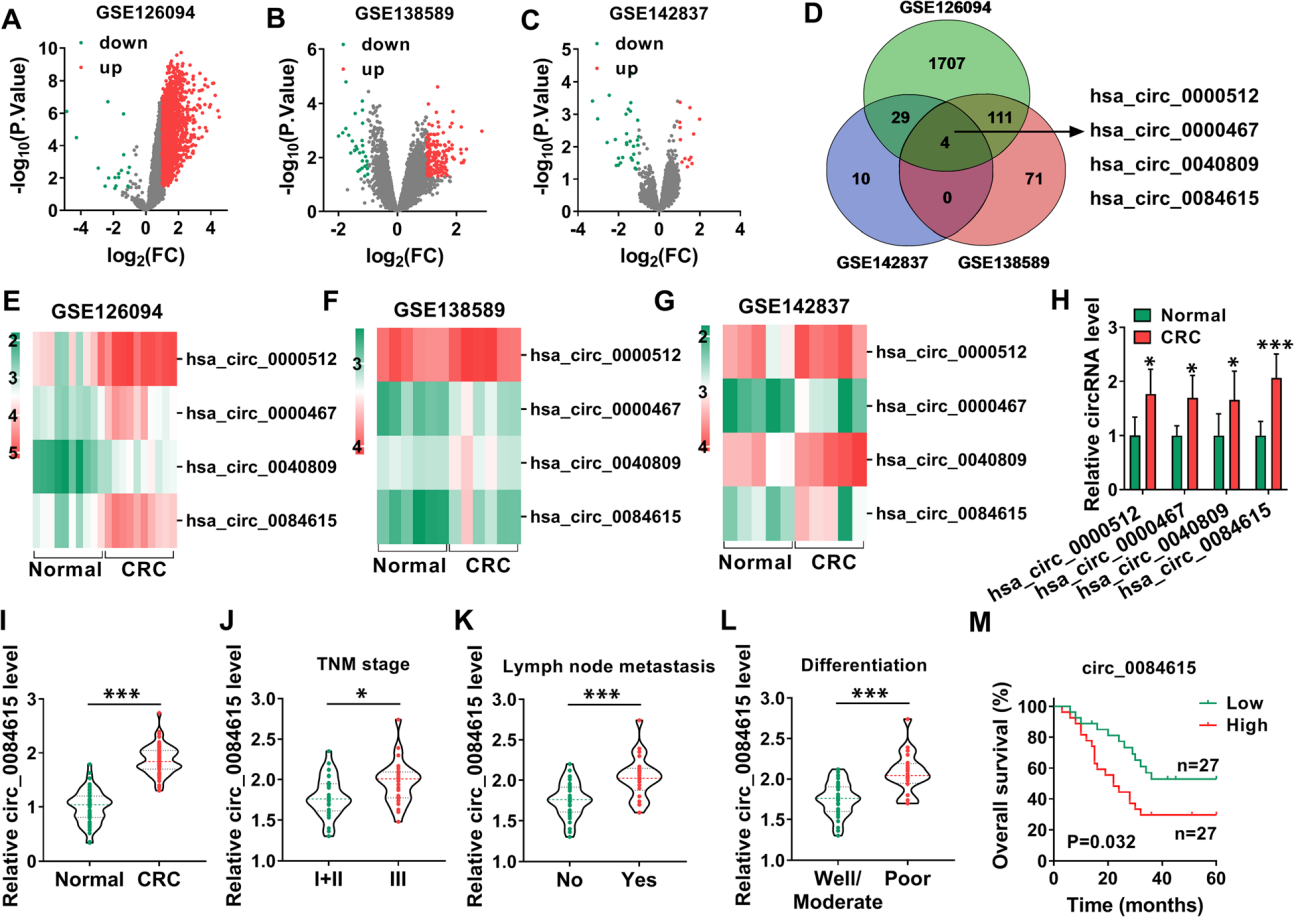
High expression of circ_0084615 in CRC tissues. (**A**-**C**) Volcano plot of differentially expressed circRNAs in CRC. (**D**) The differentially expressed circRNAs simultaneously predicted in GSE126094, GSE138589 and GSE142837. (**E**-**G**) Heatmap analysis showed the high levels of hsa_circ_0000512, hsa_circ_0000467, hsa_circ_0040809 and hsa_circ_0084615 in CRC tissues. (**H**) The levels of hsa_circ_0000512, hsa_circ_0000467, hsa_circ_0040809 and hsa_circ_0084615 in CRC tissues (*n* = 8) and normal tissues *(n* = 8) were detected by qRT-PCR assay. (**I**) The expression of circ_0084615 in CRC tissues (*n* = 54) and normal tissues (*n* = 54) was determined by qRT-PCR assay. (**J**) The expression of circ_0084615 in CRC patients at different TNM stages (29 patients at TNM stages I + II and 25 patients at stage III) was detected by qRT-PCR assay. (**K**) The expression of circ_0084615 in CRC patients with (*n* = 22) or without (*n* = 32) lymph node metastasis was detected by qRT-PCR assay. (**L**) The expression of circ_0084615 in CRC patients with well/moderate (*n* = 34) and poor differentiation (*n* = 20) was detected by qRT-PCR assay. (**M**) The overall survival of CRC patients with high or low circ_0084615 expression was analyzed. *P < 0.05, ***P < 0.001

As we observed in Fig. 1 A-C, volcano plot showed the differentially expressed circRNAs in databases GSE126094, GSE138589 and GSE142837. Among these, 4 aberrantly expressed circRNAs (hsa_circ_0000512, hsa_circ_0000467, hsa_circ_0040809 and hsa_circ_0084615) were simultaneously found in GSE126094, GSE138589 and GSE142837 (Fig. 1D). Heatmap analysis showed that hsa_circ_0000512, hsa_circ_0000467, hsa_circ_0040809 and hsa_circ_0084615 were all upregulated in CRC tissues compared to normal tissues (Fig. 1E-G). qRT-PCR showed that hsa_circ_0000512, hsa_circ_0000467, hsa_circ_0040809 and hsa_circ_0084615 were highly expressed in CRC tissues (*n* = 8) compared to corresponding control tissues (*n* = 8) (Fig. 1 H). Given the higher expression of hsa_circ_0084615 than hsa_circ_0000512, hsa_circ_0000467 and hsa_circ_0040809 in CRC tissues, circ_0084615 was selected for further study. Compared to normal tissues (*n* = 54), circ_0084615 was markedly elevated in CRC tissues (*n* = 54) (Fig. 1I). Moreover, our results showed that circ_0084615 level was increased in CRC patients in TNM stage III (*n* = 25), lymph node metastasis (*n* = 22) and poor differentiation (*n* = 20) groups compared to the patients in TNM stages I + II (*n* = 29), not lymph node metastasis (*n* = 32) and well/moderate differentiation (*n* = 34) groups (Fig. 1 J-L). Besides, the patients were divided into 2 groups (high level of circ_0084615 and low level of circ_0084615) according to the median value and high level of circ_0084615 was related to poor overall survival of CRC patients (Fig. 1 M).

### High expression of circ_0084615 in CRC cells and the identification of circ_0084615

**Fig. 2:**
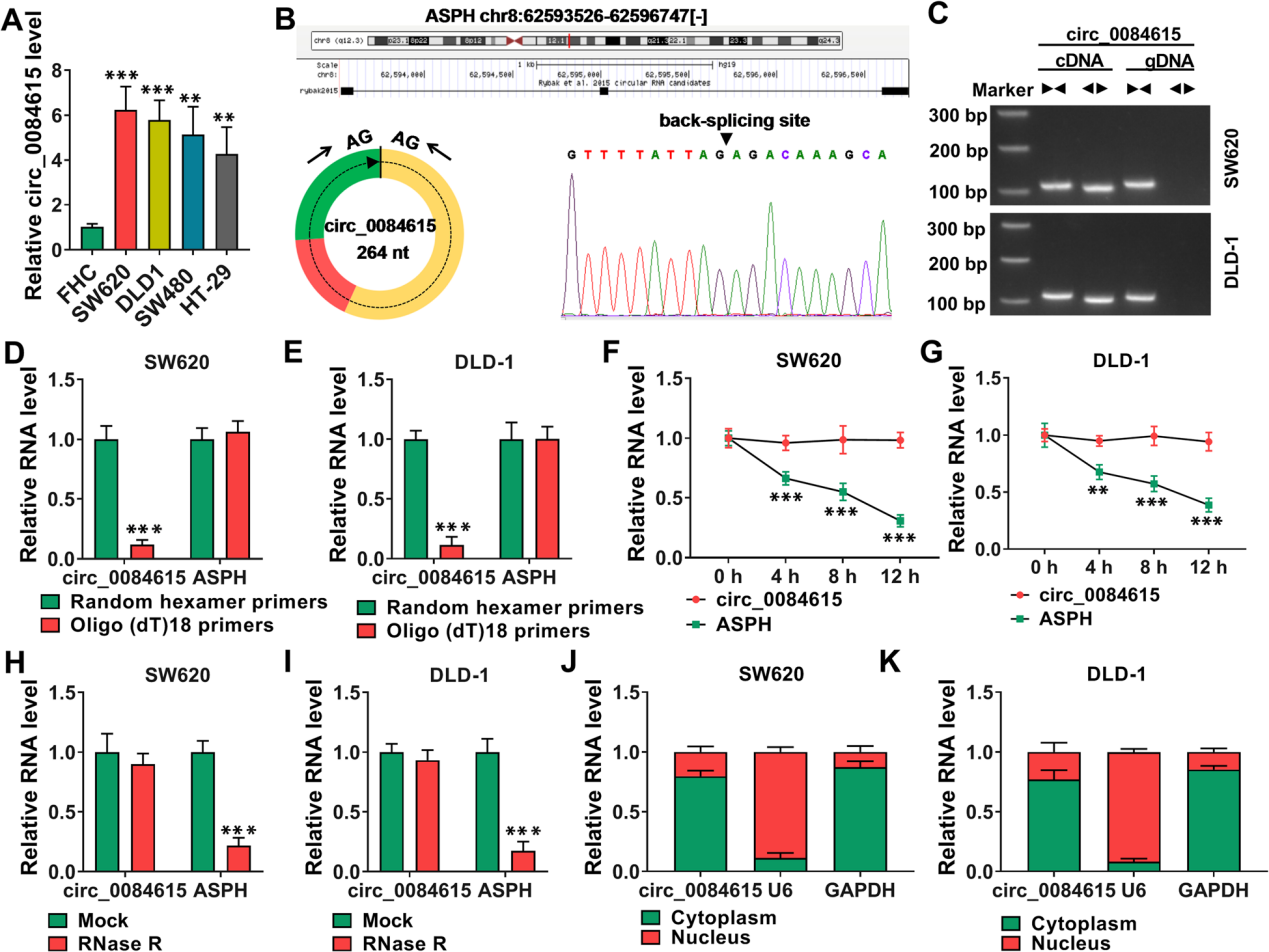
The expression of circ_0084615 in CRC cells and the features of circ_0084615. (**A**) The expression of circ_0084615 in FHC, SW620, DLD1, SW480 and HT-29 cells was detected by qRT-PCR assay. (**B**) Circ_0084615 was formed by gene ASPH. (**C**) The existence of CIRC_0084615 was detected by qRT-PCR assay with divergent or convergent primers. (**D** and **E**) The existence of circ_0084615 in the reverse transcription products using random hexamer primers and oligo (dT)18 primers. (**F** and **G**) The levels of circ_0084615 and ASPH in SW620 and DLD-1 cells treated with Act D were examined with qRT-PCR assay. (**H** and **I**) The levels of circ_0084615 and ASPH in SW620 and DLD-1 cells treated with or without RNase R were detected by qRT-PCR assay. (**J** and **K**) The expression of circ_0084615 in the cytoplasm and nucleus of SW620 and DLD-1 cells was analyzed with subcellular fraction analysis. **P < 0.01, ***P < 0.001

As shown in Fig. 2A, circ_0084615 was notably increased in SW620, DLD1, SW480 and HT-29 cells compared to FHC cells. Circ_0084615 located at chr8: 62593526–62596747 and was formed by the exons 2–4 of ASPH with a mature length of 264 nt (Fig. 2B). Then, divergent primers were designed to amplify circ_0084615 and convergent primers were designed to amplify ASPH. We found that circ_0084615 amplification product was only detectable in cDNA by divergent primers and ASPH was amplified in both cDNA and genomic DNA (gDNA) by convergent primers (Fig. 2 C). Due to the lack of 3’ poly a tail, we analyzed the existence of circ_0084615 in the reverse transcription products by using random hexamer primers and oligo (dT)18 primers. We found that oligo (dT)18 primers barely amplified circ_0084615 (Fig. 2D and E). Act D assay showed that circ_0084615 possessed a longer half-life than ASPH (Fig. 2 F and G). RNase R assay showed that ASPH was digested by RNase R, while circ_0084615 was resistant to RNase R (Fig. 2 H and I). In addition, it was found that circ_0084615 mainly enriched in the cytoplasm instead of the nucleus in SE620 and DLD-1 cells (Fig. 2 J and K).

### Effects of circ_0084615 on CRC cell proliferation, migration, invasion and angiogenesis

**Fig. 3:**
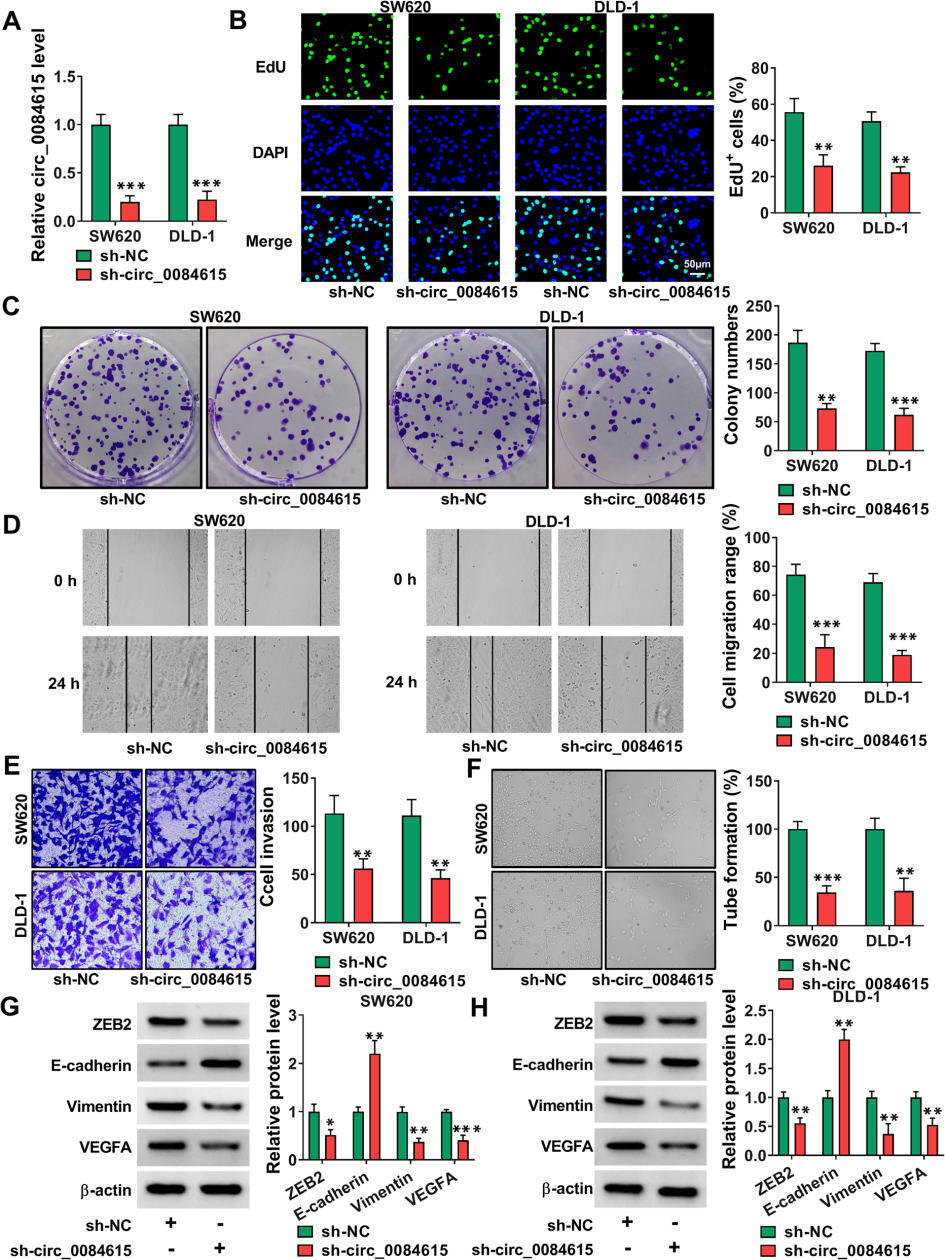
Circ_0084615 interference suppressed cell proliferation, migration, invasion and angiogenesis in CRC cells. SW620 and DLD-1 cells were introduced with sh-NC or sh-circ_0084615. (**A**) The expression of circ_0084615 in SW620 and DLD-1 cells was examined by qRT-PCR assay. (**B**-**F**) The proliferation, colony formation, migration, invasion and angiogenesis of SW620 and DLD-1 cells were assessed by EdU assay, colony formation assay, wound-healing assay, transwell assay and tube formation assay, respectively. (**G** and **H**) The protein levels of ZEB2, E-cadherin, Vimentin and VEGFA in SW620 and DLD-1 cells were measured via western blot assay. *P < 0.05, **P < 0.01, ***P < 0.001

To clarify the potential roles of circ_0084615 in CRC progression, loss-of-function study was conducted by transfecting sh-circ_0084615 into SW620 and DLD-1 cells. As a result, sh-circ_0084615 transfection led to a distinct reduction in circ_0084615 expression in SW620 and DLD-1 cells compared to sh-NC control groups (Fig. 3 A). EdU assay and colony formation assay showed that circ_0084615 silencing markedly suppressed the proliferation and colony formation of SW620 and DLD-1 cells compared to sh-NC groups (Fig. 3B and C). As demonstrated by wound healing assay and transwell assay, the migration and invasion of SW620 and DLD-1 cells were restrained by decreasing circ_0084615 in comparison with sh-NC control groups (Fig. 3D and E). Tube formation assay indicated that circ_0084615 knockdown repressed the angiogenesis ability of HUVECs compared to control groups (Fig. 3 F). Besides, the protein levels of epithelial-mesenchymal transition (EMT)-related markers (ZEB2, E-cadherin and Vimentin) and VEGFA were measured. The results presented that circ_0084615 deficiency decreased ZEB2, Vimentin and VEGFA protein levels and increased E-cadherin protein level in SW620 and DLD-1 cells (Fig. 3G and H). Collectively, circ_0084615 silencing inhibited CRC cell growth, metastasis and angiogenesis.

### Circ_0084615 directly targeted miR-599

**Fig. 4:**
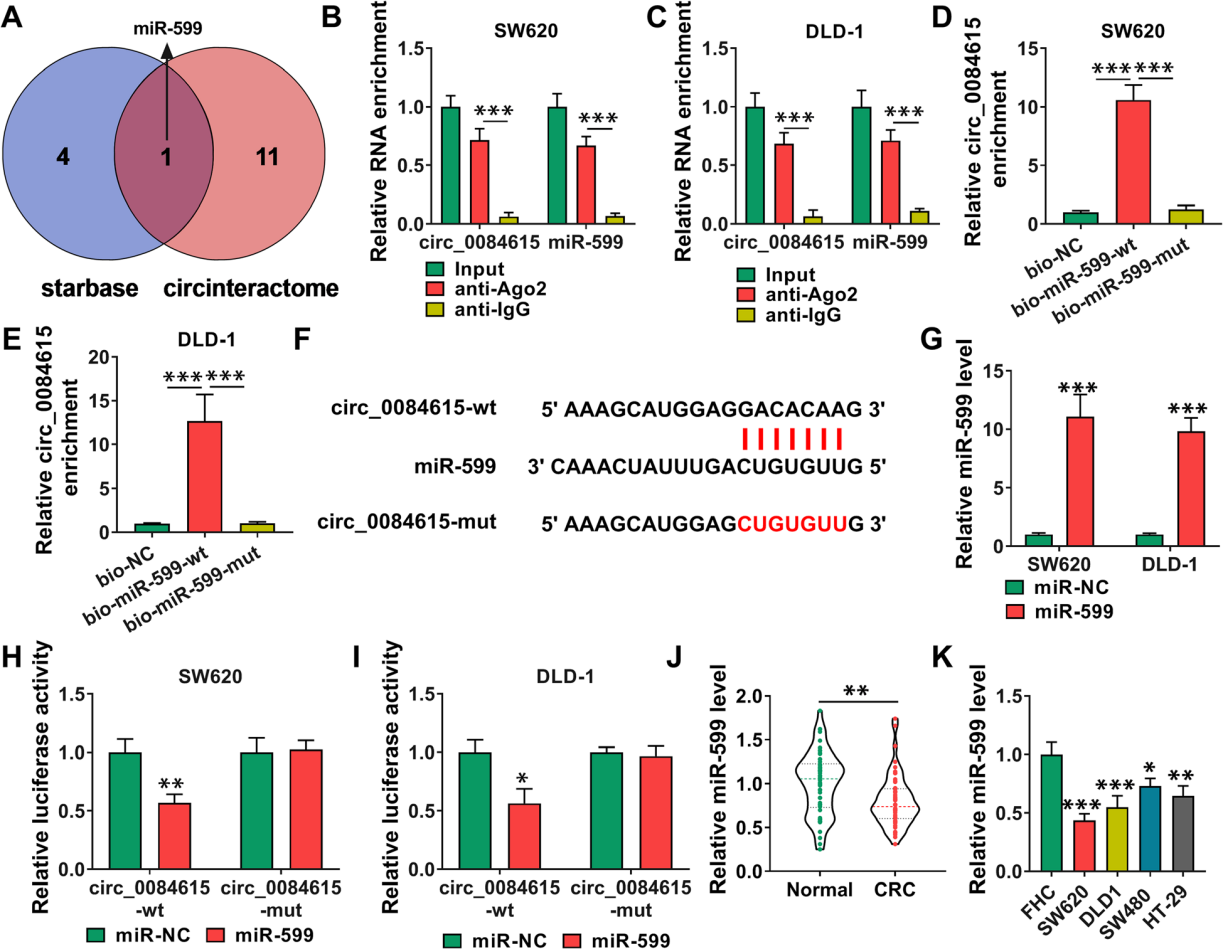
Circ_0084615 bound to miR-599 in CRC cells. (**A**) The targets of circ_0084615 were predicted by starbase and circinteractome. (**B**-**E**) The relationship between circ_0084615 and miR-599 was analyzed by RIP and RNA pull-down assays. (**F**) The complementary sequences between circ_0084615 and miR-599. (**G**) The expression of miR-599 in SW620 and DLD-1 cells transfected with miR-NC or miR-599 was detected by qRT-PCR assay. (**H** and **I**) The interaction between circ_0084615 and miR-599 was examined by dual-luciferase reporter assay. (**J** and **K**) The expression of miR-599 in CRC tissues and cells was determined by qRT-PCR assay. *P < 0.05, **P < 0.01, ***P < 0.001

Through analyzing starbase (http://starbase.sysu.edu.cn/starbase2/) and circinteractome (https://circinteractome.irp.nia.nih.gov/), miR-599 was found to be the target of circ_0084615 (Fig. 4 A). RIP assay indicated that circ_0084615 and miR-599 were significantly enriched in the immunoprecipitated complexes of anti-Ago2 groups compared to anti-IgG groups (Fig. 4B and C). RNA pull-down assay showed that bio-miR-599-wt pull down more circ_0084615 than bio-NC and bio-miR-599-mut control groups (Fig. 4D and E). The binding sites between circ_0084615 and miR-599 were exhibited in Fig. 4 F. MiR-599 mimic transfection led to an elevation of miR-599 expression in SW620 and DLD-1 cells (Fig. 4G). Dual-luciferase reporter assay showed that the luciferase intensity of circ_0084615-wt was repressed in SW620 and DLD-1 cells with miR-599 overexpression, but the luciferase intensity of circ_0084615-mut was not changed (Fig. 4 H and I). Expectedly, miR-599 was lowly expressed in CRC tissues and cells relative to normal tissues and cells (Fig. 4 J and K). All these results indicated that miR-599 was a target of circ_0084615.

### Circ_0084615 regulated CRC cell growth, metastasis and angiogenesis by targeting miR-599

**Fig. 5:**
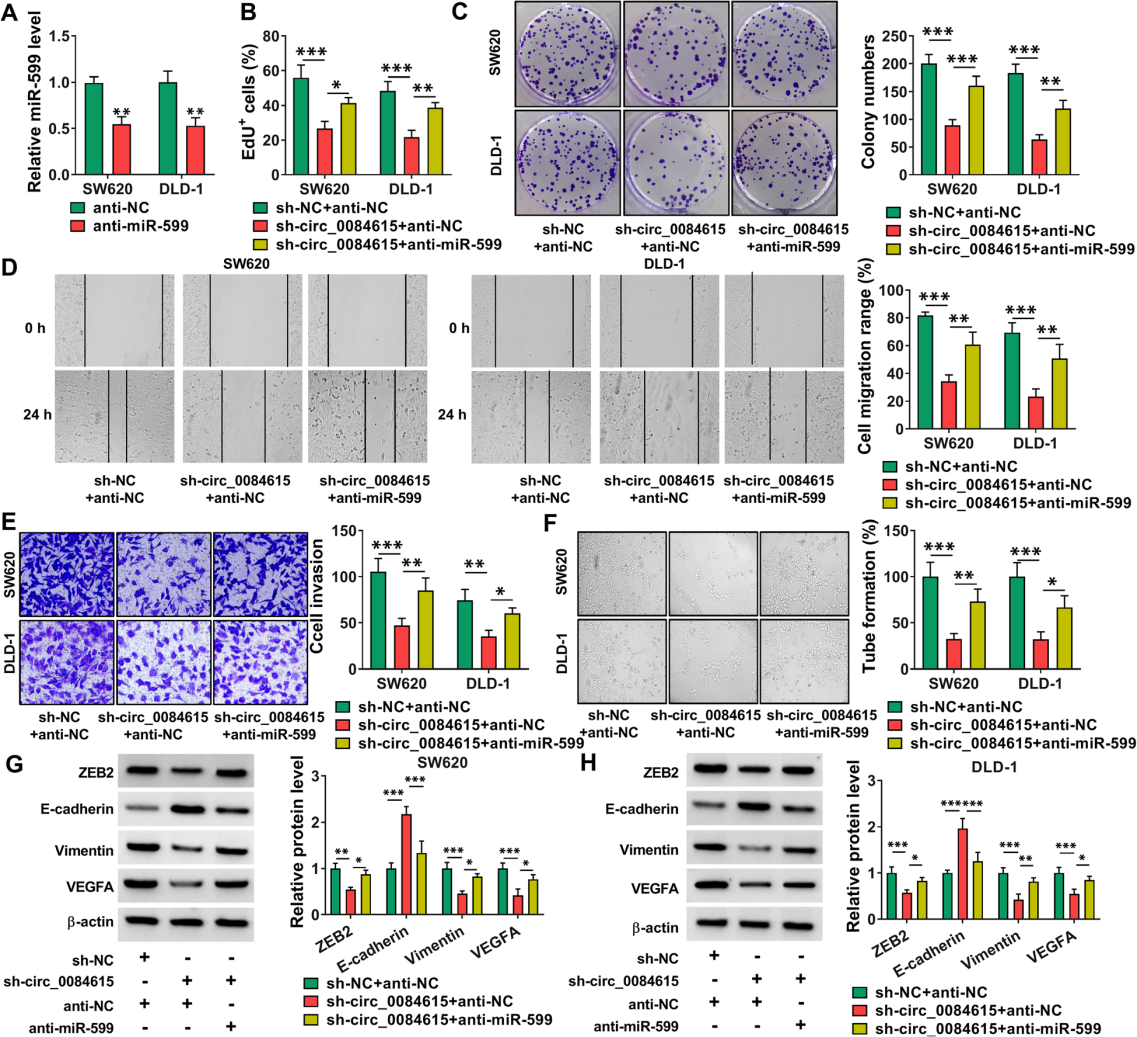
Inhibition of miR-599 rescued the impacts of circ_0084615 knockdown on CRC cell malignant behaviors. (**A**) The expression of miR-599 in SW620 and DLD-1 cells transfected with anti-NC or anti-miR-599 was detected by qRT-PCR assay. (**B**-**H**) SW620 and DLD-1 cells were transfected with sh-NC + anti-NC, sh-circ_0084615 + anti-NC or sh-circ_0084615 + anti-miR-599. (**B**-**F**) The proliferation, colony formation, migration, invasion and angiogenesis of SW620 and DLD-1 cells were evaluated via EdU assay, colony formation assay, wound-healing assay, transwell assay and tube formation assay, respectively. (**G** and **H**) The protein levels of ZEB2, E-cadherin, Vimentin and VEGFA in SW620 and DLD-1 cells were measured via western blot assay. *P < 0.05, **P < 0.01, ***P < 0.001

As presented in Fig. 5 A, anti-miR-599 transfection reduced miR-599 level in SW620 and DLD-1 cells compared to anti-miR-599. Then sh-NC + anti-NC, sh-circ_0084615 + anti-NC or sh-circ_0084615 + anti-miR-599 was transfected into SW620 and DLD-1 cells to explore the relationship between circ_0084615 and miR-599 in regulating CRC progression. EdU assay and colony formation assay showed that miR-599 inhibition reversed circ_0084615 knockdown-mediated suppressive effects on cell proliferation and colony formation in SW620 and DLD-1 cells (Fig. 5B and C). Wound-healing assay and transwell assay indicated that circ_0084615 silencing repressed the migration and invasion of SW620 and DLD-1 cells, while these effects were partially ameliorated by reducing miR-599 (Fig. 5D and E). Tube formation assay showed that circ_0084615 interference hampered the tube formation ability of HUVECs, with miR-599 downregulation abated the effect (Fig. 5 F). Furthermore, the effects of circ_0084615 knockdown on ZEB2, E-cadherin, Vimentin and VEGFA levels in SW620 and DLD-1 cells were reversed by decreasing miR-599 (Fig. 5G and H). Taken together, circ_0084615 knockdown inhibited the malignancy of CRC cells by targeting miR-599.

### ONECUT2 was the target gene of miR-599

**Fig. 6:**
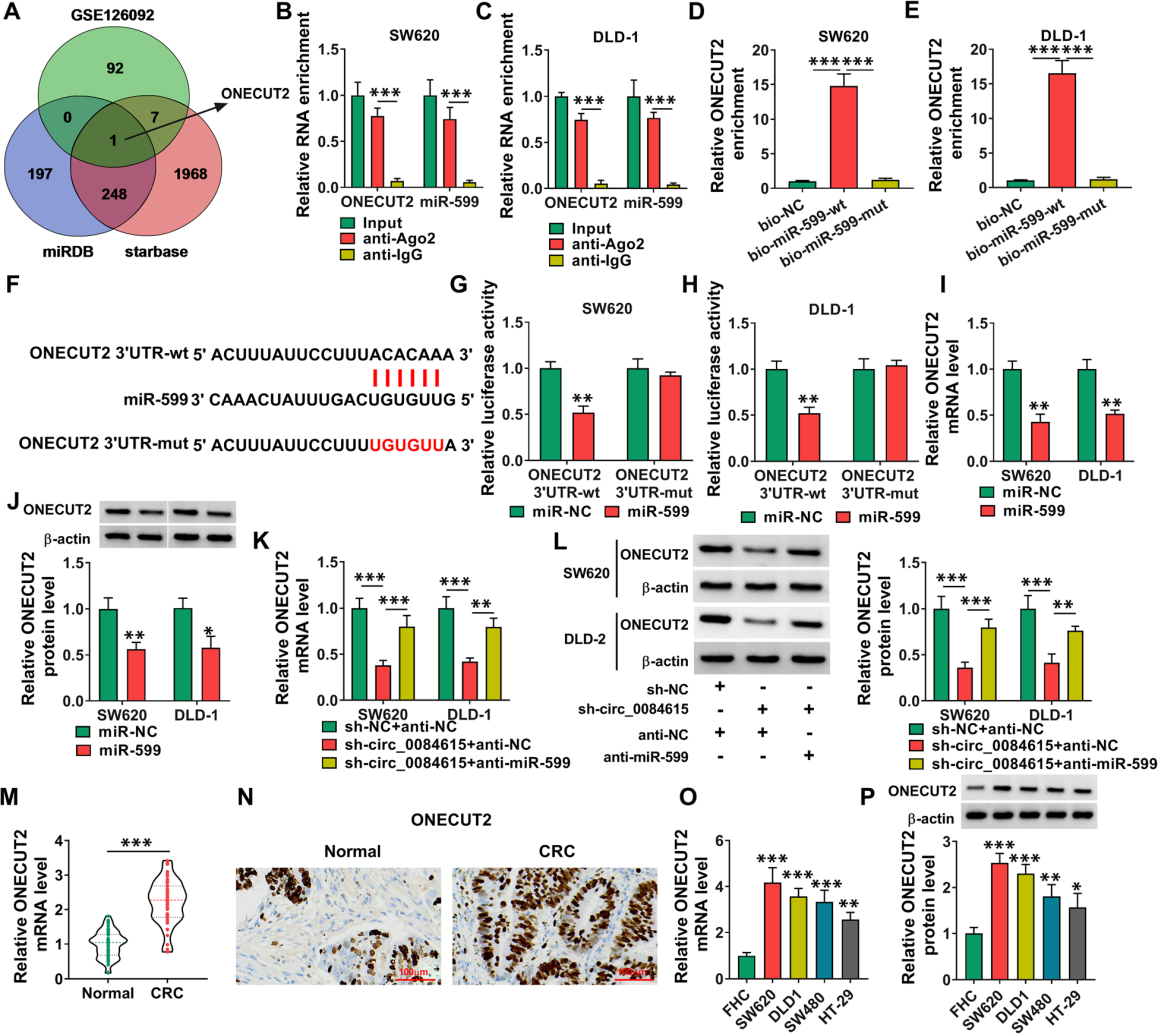
MiR-599 directly interacted with ONECUT2. (**A**) ONECUT2 was predicted to be the target gene of miR-599. (**B**-**E**) The combination between miR-599 and ONECUT2 was assessed by RIP and RNA pull down assays. (**F**) The binding sites between ONECUT2 and miR-599. (**G** and **H**) The combination between miR-599 and ONECUT2 was analyzed by dual-luciferase reporter assay. (**I** and **G**) The mRNA and protein levels of ONECUT2 in SW620 and DLD-1 cells transfected with miR-NC or miR-599 were measured by qRT-PCR assay or western blot assay. (**K** and **L**) The mRNA and protein levels of ONECUT2 in SW620 and DLD-1 cells transfected with sh-NC + anti-NC, sh-circ_0084615 + anti-NC or sh-circ_0084615 + anti-miR-599 were measured by qRT-PCR assay or western blot assay. (**M** and **N**) The expression of ONECUT2 in CRC tissues and normal tissues was examined with qRT-PCR and IHC. (**O** and **P**) The mRNA and protein levels of ONECUT2 in FHC, SW620, DLD1, SW480 and HT-29 cells were measured by qRT-PCR assay or western blot assay. *P < 0.05, **P < 0.01, ***P < 0.001

Through analyzing GSE12609, we selected 100 upregulated genes in CRC and found that only ONECUT2 was simultaneously predicted to be the target gene of miR-599 by starbase and miRDB (http://mirdb.org/) (Fig. 6 A). RIP assay showed that the enrichment of ONECUT2 and miR-599 was increased by anti-Ago2 RIP compared to anti-IgG RIP groups (Fig. 6B and C). RNA pull down assay showed that ONECUT2 was enriched by bio-miR-599-wt compared to bio-NC or bio-miR-599-mut (Fig. 6D and E). As shown in Fig. 6 F, ONECUT2 contained miR-599 binding sites. Then dual-luciferase reporter assay indicated that miR-599 elevation repressed the luciferase activity of ONECUT2 3’UTR-wt in SW620 and DLD-1 cells but did not affect the luciferase activity of ONECUT2 3’UTR-mut (Fig. 6G and H). These results demonstrated the interaction between ONECUT2 and miR-599. Moreover, miR-599 overexpression markedly decreased the mRNA and protein levels of ONECUT2 in both SW620 and DLD-1 cells (Fig. 6I and J). Circ_0084615 knockdown decreased the mRNA and protein levels of ONECUT2 in SW620 and DLD-1 cells, whereas miR-599 inhibition reversed the impacts (Fig. 6 K and L). Indeed, qRT-PCR and IHC assay showed that ONECUT2 level was increased in tumor tissues compared to normal tissues (Fig. 6 M and N). In addition, ONECUT2 mRNA and protein levels were increased in CRC cells compared to FHC cells (Fig. 6O and P). Collectively, circ_0084615 altered ONECUT2 expression by targeting miR-599.

### Overexpression of miR-599 suppressed cell proliferation, migration, invasion and angiogenesis in CRC cells

**Fig. 7:**
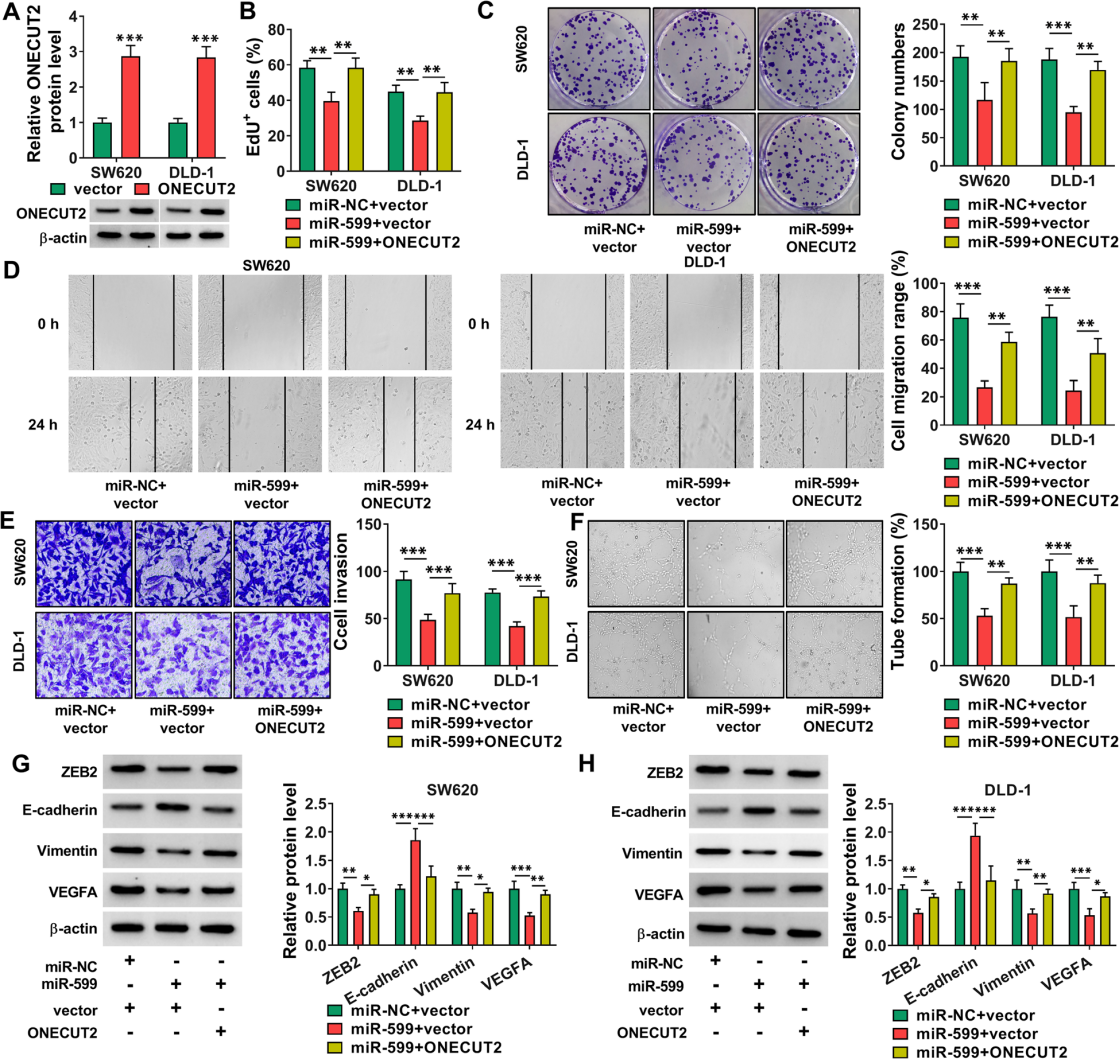
MiR-599 overexpression altered CRC cell growth, motility and angiogenesis by binding to ONECUT2. (**A**) The protein level of ONECUT2 in SW620 and DLD-1 cells transfected with ONECUT2 or vector was measured via western blot assay. (**B**-**H**) SW620 and DLD-1 cells were transfected with miR-NC + vector, miR-599 + vector or miR-599 + ONECUT2. (**B**-**F**) The proliferation, colony formation, migration, invasion and angiogenesis of SW620 and DLD-1 cells were investigated by EdU assay, colony formation assay, wound healing assay, transwell assay and tube formation assay, respectively. (**G** and **H**) The protein levels of ZEB2, E-cadherin, Vimentin and VEGFA in SW620 and DLD-1 cells were measured via western blot assay. *P < 0.05, **P < 0.01, ***P < 0.001

The transfection of ONECUT2 overexpression resulted in a marked elevation of ONECUT2 protein level in SW620 and DLD-1 cells (Fig. 7 A). Next, whether miR-599 could regulate CRC cell progression by targeting ONECUT2 was investigated. As illustrated by EdU assay and colony formation assay, miR-599 overexpression restrained the ability of SW620 and DLD-1 cells to proliferate, while ONECUT2 elevation rescued the effect (Fig. 7B and C). Overexpression of miR-599 impeded the migration and invasion of SW620 and DLD-1 cells, with ONECUT2 upregulation reversed the effects (Fig. 7D and E). Tube formation assay showed that miR-599 overexpression inhibited the tube formation ability of HUVECs, while the impact was weakened by elevating ONECUT2 (Fig. 7 F). Additionally, miR-599 enhancement reduced ZEB2, Vimentin and VEGFA levels and elevated E-cadherin levels in SW620 and DLD-1 cells, with ONECUT2 overexpression abated the impacts (Fig. 7G and H). To sum up, miR-599 overexpression inhibited the malignant behaviors of CRC cells by targeting ONECUT2.

### EIF4A3 induced circ_0084615 expression in CRC cells

**Fig. 8:**
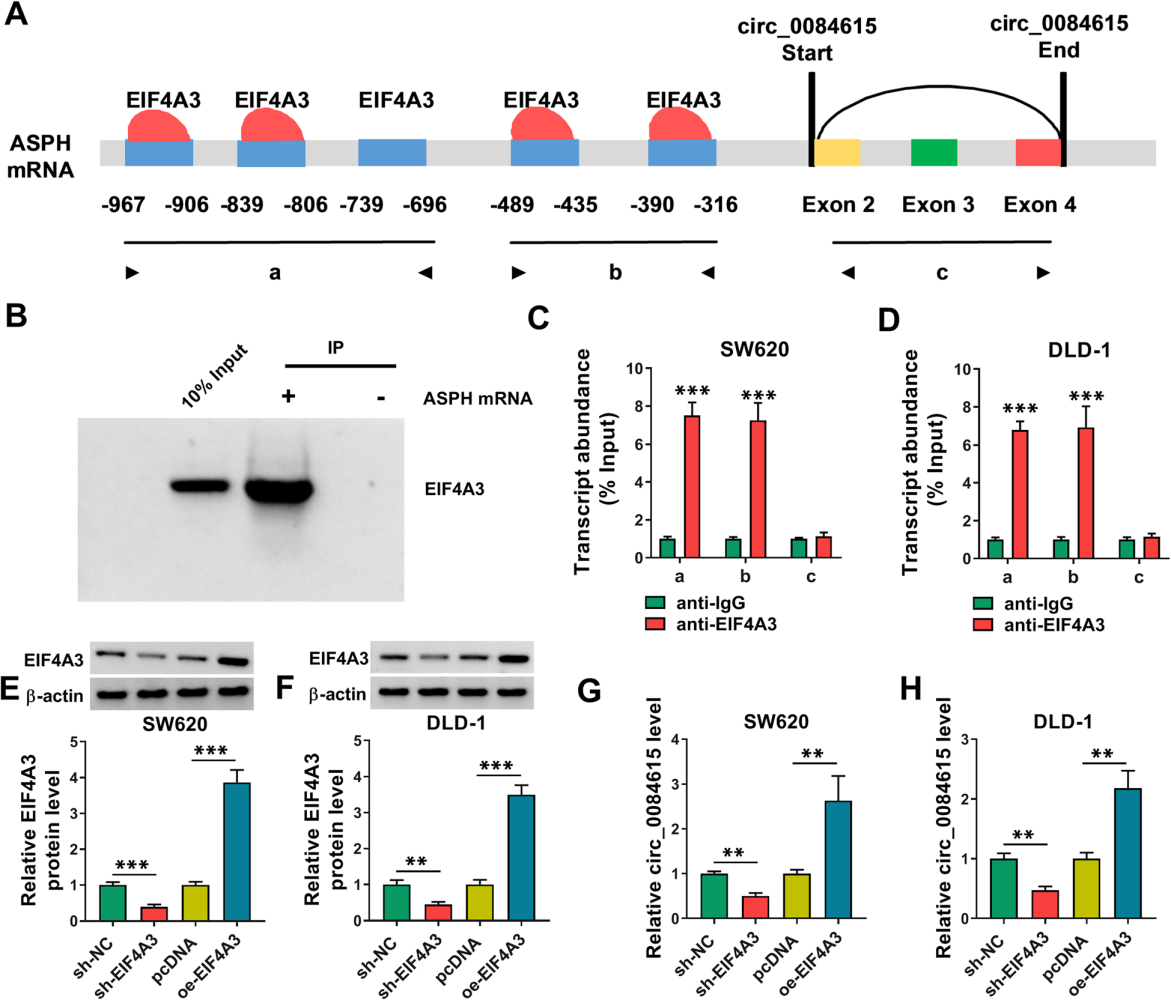
EIF4A3 regulated circ_0084615 expression. (**A**) The binding sites of EIF4A3 in the flanking sequences of the ASPH1 mRNA transcript were predicted. (**B**-**D**) RIP assay demonstrated the binding between ASPH and EIF4A3. (**E**-**H**) The levels of EIF4A3 and circ_0084615 in SW620 and DLD-1 cells transfected with sh-NC, sh-EIF4A3, pcDNA or oe-EIF4A3 was measured through western blot assay or qRT-PCR assay. **P < 0.01, ***P < 0.001

It has been reported that some RNA-binding proteins can bind to the flanking of the intron sequence of circRNAs and influence circRNA production [19]. EIF4A3 is a regulator of exon splicing [20]. Through analyzing circinteractome (https://circinteractome.irp.nia.nih.gov/), we found that EIF4A3 had 5 binding sites on the upstream region of ASPH mRNA transcript (Fig. 8 A). RIP assay showed that EIF4A3 could bind to ASPH mRNA via the binding regions a and b but not circ_0084615 (region c) (Fig. 8B-D). These results indicated that EIF4A3 could bind to the flanking sequences of circ_0084615 through the potential binding sites. Furthermore, sh-EIF4A3 or oe-EIF4A3 was transfected into SW620 and DLD-1 cells to reduce or elevate EIF4A3 expression, which were verified by western blot assay (Fig. 8E and F). Of note, our results showed that EIF4A3 knockdown reduced circ_0084615 level and EIF4A3 overexpression increased circ_0084615 expression in SW620 and DLD-1 cells (Fig. 8G and H). Taken together, EIF4A3 positively regulated circ_0084615 expression.

### Circ_0084615 silencing hampered tumor growth in vivo

**Fig. 9:**
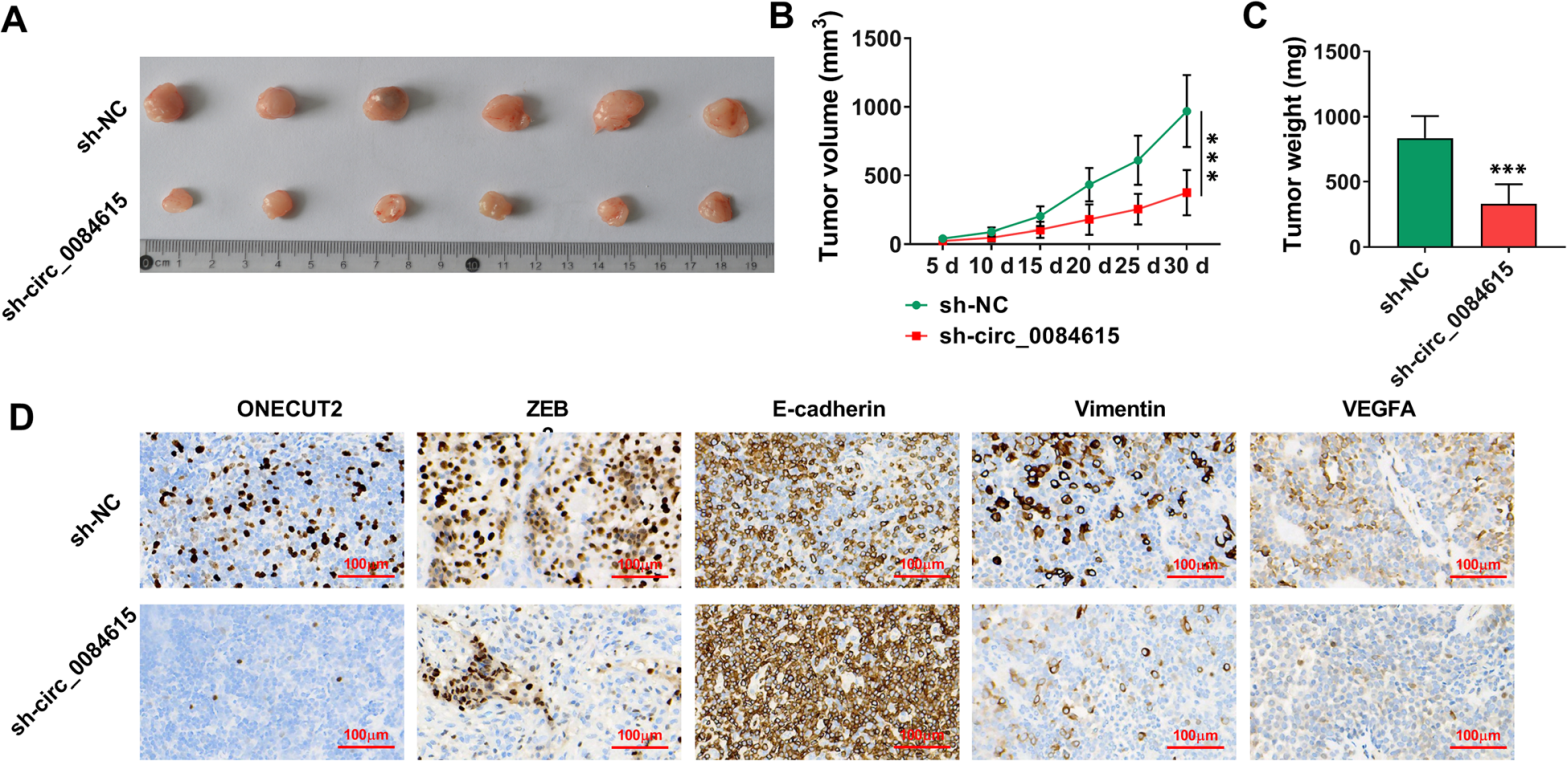
Deficiency of circ_0084615 suppressed tumor formation in vivo. (**A**-**C**) Tumor volume and tumor weight were examined. (**D**) The levels of ONECUT2, ZEB2, E-cadherin, Vimentin and VEGFA in xenograft tumors were examined with IHC assay. ***P < 0.001

Subsequently, the roles of circ_0084615 in tumor growth were investigated. The results of murine xenograft model assay showed that the mice with circ_0084615 silencing possessed the reduced tumor volume and tumor weight compared to control groups (Fig. 9 A-C). IHC assay showed that ONECUT2, ZEB2, Vimentin and VEGFA levels were reduced and E-cadherin level was elevated in the xenograft tumors in sh-circ_0084615 groups compared to sh-NC groups (Fig. 9D). These results indicated that circ_0084615 played a positive role in tumor growth *in vivo*.

## Discussion

Accumulating evidence has revealed that the dysregulation of circRNAs are linked to CRC progression and may be used as the potential diagnostic biomarkers [21]. The current study unveiled the functions of circ_0084615 in CRC for the first time. Moreover, the novel regulatory pathway circ_0084615/miR-599/ONECUT2 in CRC development was discovered.

In CRC, diverse circRNAs, such as circIFT80 [22], circ_0053277 [23], circ-ITGA7 [24], circ_001917 [25], have been reported to mediate CRC development via altering tumor cell growth, migration, EMT and angiogenesis. Even though, the effects of circ_0084615 on CRC are unclear. The CRC-related GEO databases showed the abnormal upregulation of circ_0084615 in CRC, thus, we further explored the exact roles of circ_0084615 in CRC progression. As a result, circ_0084615 was found to be overexpressed in CRC and was linked to TNM stages, lymph node metastasis, poor differentiation and dismal overall survival in CRC patients. These results suggested that the potential of circ_0084615 in acting as diagnostic and prognostic markers for CRC. Functionally, circ_0084615 interference restrained the proliferation, migration, invasion and angiogenesis in CRC cells *in vitro* and blocked tumor formation *in vivo*. Our results also showed that EMT-related markers ZEB2 and Vimentin were reduced and E-cadherin was elevated in CRC cells with circ_0084615 silencing. VEGFA is defined as a key mediator in angiogenesis and invasion of tumors [26]. Thus, we determined the influence of circ_0084615 on VEGFA expression and found that circ_0084615 knockdown reduced VEGFA level in CRC cells.

Thereafter, we elucidated the potential mechanism of circ_0084615 in CRC. We demonstrated that circ_0084615 served as the sponge for miR-599, which directly bound to ONECUT2. Yu et al. unveiled that miR-599 overexpression repressed CRC cell viability and migration via targeting ARPP19 [16]. Herein, miR-599 was reduced in CRC and repressed CRC cell growth, metastasis and angiogenesis, which was consistent with the former report. MiR-599 inhibition abrogated circ_0084615 knockdown-mediated effects on CRC cell malignant behaviors, suggesting that circ_0084615 regulated CRC cell development via targeting miR-599. Sun et al. claimed that ONECUT2 could be targeted by miR-429 to alter CRC cell proliferation and invasion [18]. However, ONECUT2 was demonstrated to be the target gene of miR-599 for the first time. ONECUT2 overexpression abated the effects of miR-599 on CRC cell malignant behaviors.

It has been reported that EIF4A3 is a modulator in RNA splicing [27]. Moreover, EIF4A3 can bind to MMP9 [20], BNIP3 [28]or ASAP1 [29] to induce circMMP9, circ-BNIP3 or circASAP1 expression. Herein, it was found that EIF4A3 induced circ_0084615 expression by binding to ASPH transcript.

## Conclusions

**Fig. 10:**
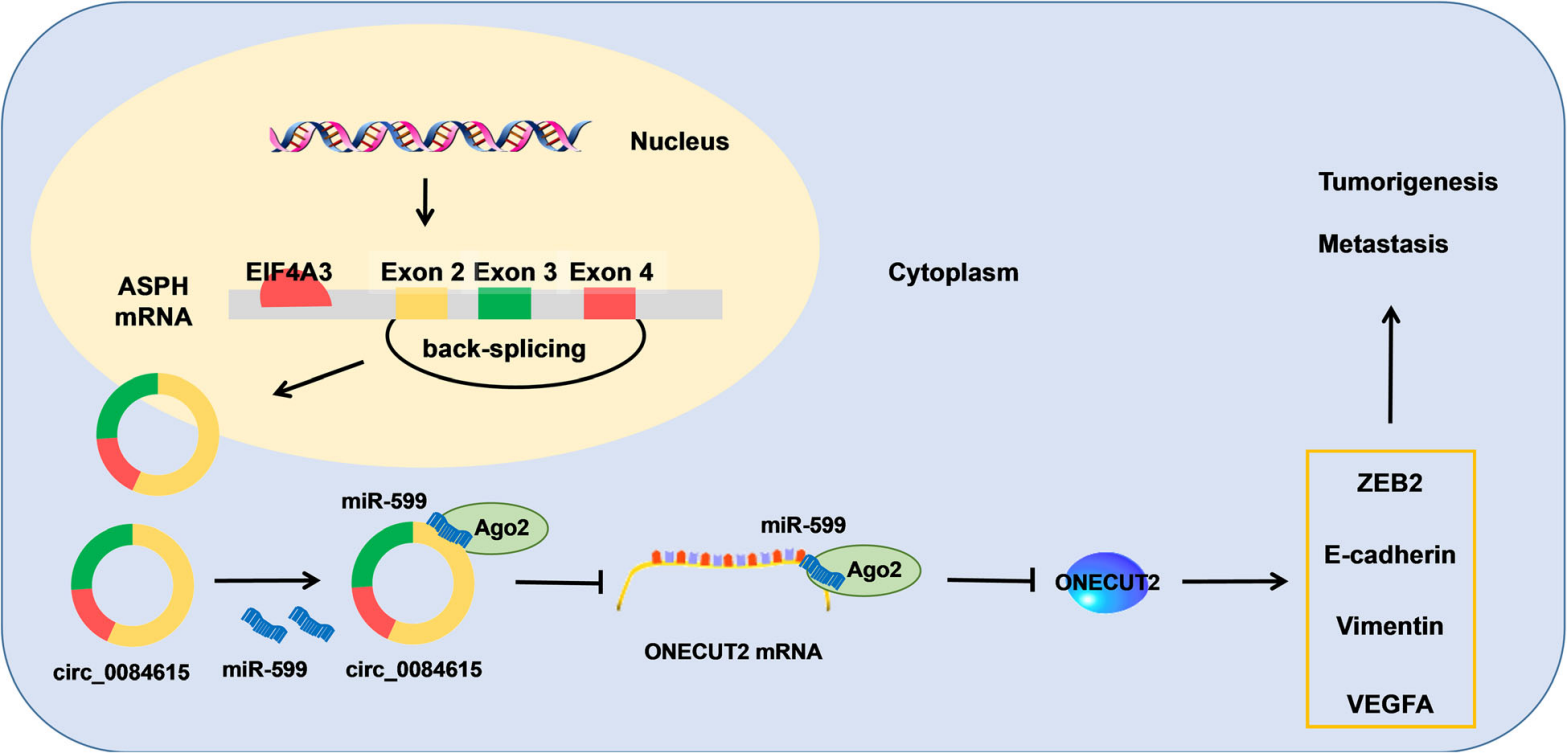
Schematic diagram of EIF4A3-mediated circ_0084615 in regulating CRC tumorigenesis and metastasis

Taken together, EIF4A3-induced circ_0084615 promoted CRC tumorigenesis and CRC cell metastasis by altering miR-599/ONECUT2 pathway (Fig. 10). Our study might provide a potential target for the diagnosis, prognosis and therapy for CRC.

## Supplementary Information

**Additional file 1.**

**Additional file 2.**

## Data Availability

Please contact the correspondence author for the data request.

## Abbreviations

CRC: colorectal cancer
qRT-PCR: Quantitative real-time polymerase chain reaction
IHC: immunohistochemistry
ASPH: aspartate beta-hydroxylase
VEGFA: vascular endothelial growth factor A
Act D: Actinomycin D
EIF4A3: eukaryotic translation initiation factor 4A3
RIP: RNA immunoprecipitation
