# Supplementary figures and images for: EIF4A3-induced circ_0084615 contributes to the progression of colorectal cancer via miR-599/ONECUT2 pathway
Source: J Exp Clin Cancer Res. 2021 Jul 12;40:227. doi: 10.1186/s13046-021-02029-y (PMC8273970; doi:10.1186/s13046-021-02029-y)

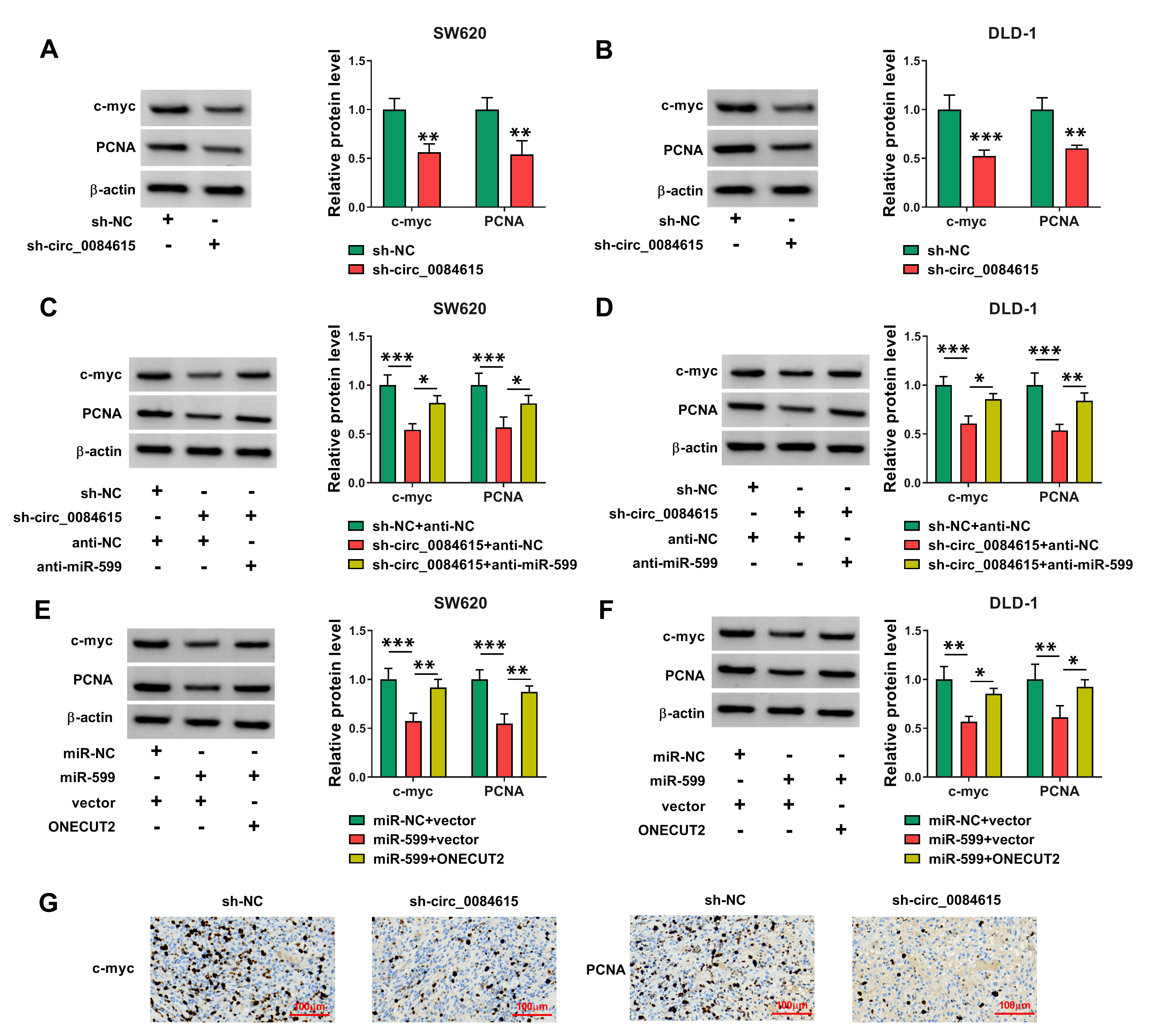

Supplement: Supplementary file 1 — Additional file 1. [file 13046_2021_2029_MOESM1_ESM.tif]

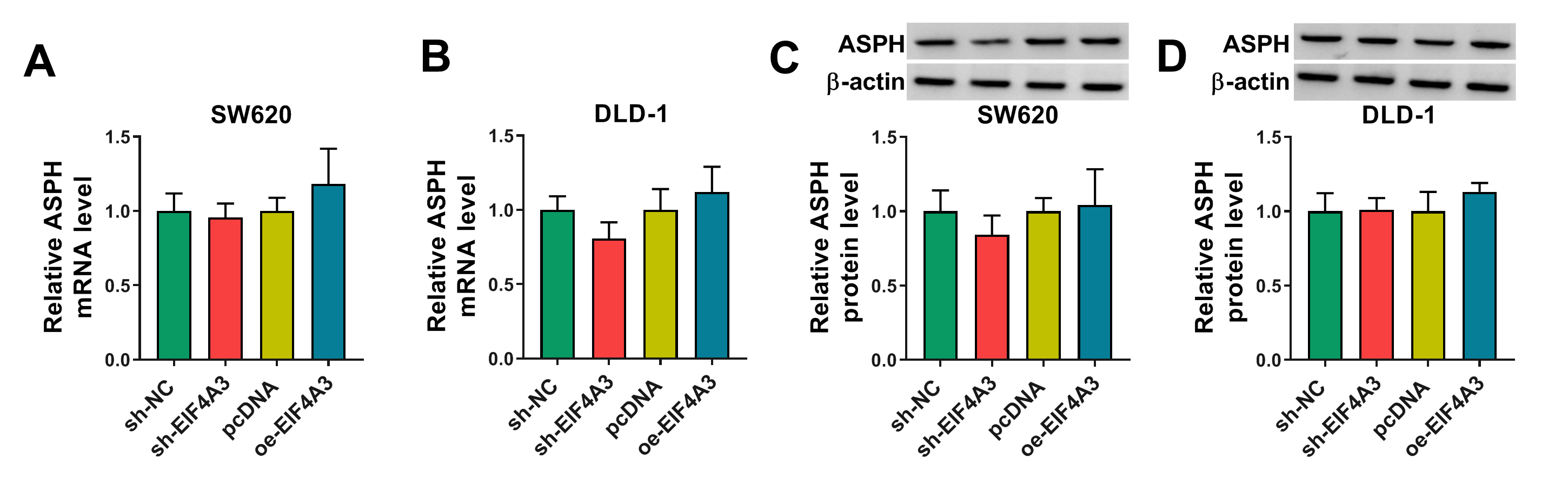

Supplement: Supplementary file 2 — Additional file 2. [file 13046_2021_2029_MOESM2_ESM.tif]
